# Evaluating the Role of Sinus Surgery on the Non-diseased Contralateral Side in Patients With Unilateral Sinus Disease: A Systematic Review

**DOI:** 10.7759/cureus.90160

**Published:** 2025-08-15

**Authors:** Noora Al-Hail, Ahmad Shaikh, Hamad Al Saey

**Affiliations:** 1 Medical Education, Otorhinolaryngology - Head and Neck Surgery (ENT), Hamad Medical Corporation, Doha, QAT; 2 Otorhinolaryngology - Head and Neck Surgery (ENT), Hamad Medical Corporation, Doha, QAT

**Keywords:** contralateral side, functional endoscopic sinus surgery (fess), inflammation, recurrence, sinusitis, unilateral sinus disease (usd)

## Abstract

Functional endoscopy sinus surgery (FESS) is a frequently employed surgical technique to treat patients with sinus diseases. However, limited studies are available that discuss the impact of sinus surgery on the non-diseased contralateral side. The present review was conducted to examine existing literature regarding the role of sinus surgery on the non-diseased contralateral side in patients with unilateral sinus disease (USD). We conducted a comprehensive systematic review using the Preferred Reporting Items for Systematic Reviews and Meta-analyses (PRISMA) guidelines. Relevant literature was searched from different electronic databases, including PubMed, Scopus, Cochrane Library, ScienceDirect, and Google Scholar, until February 2025. Studies' screening, selection, quality assessment, grading for certainty of evidence, and data extraction were done by two independent reviewers, and any disagreement was resolved by discussion. Results were integrated into tables and synthesized through a narrative summary. A total of 10 studies (N = 1,233 patients), aged 27.5-64.4 years, with a higher ratio of female participants, were included in the present review. Different pathological conditions were reported in selected studies, including chronic rhinosinusitis (CRS) alone, CRS with nasal polyposis (NP), antrochoanal polyp (ACP), fungus ball, benign tumor, malignant neoplasia, NP alone, acute or chronic unilateral sinusitis, and unilateral allergic fungal rhinosinusitis (AFRS). Most studies observed significant patient improvement, with a high success rate in improving symptoms such as nasal obstruction and postnasal drainage. However, studies also reported disease recurrence on the non-diseased contralateral side. Multiple medical interventions typically involve β-lactam antibiotics, decongestants, mucolytics, and nasal steroids to reduce inflammation and control the recurrence of diseases. Most studies showed a low risk of bias. In addition, no publication bias was observed. No serious concern was found in the reporting of these observational studies and found low risk of bias among the studies. A high level of certainty of evidence was observed. The present findings highlight that unilateral FESS improves one side of sinusitis; however, it gradually affects the non-diseased contralateral side, suggesting the importance of patient counseling and resulting in disease events on the contralateral side. Further, multicentre studies with large sample sizes are needed to validate these findings. Our study explores the potential benefits of operating on the contralateral side in cases of USD to prevent future recurrence on the previously uninvolved side.

## Introduction and background

Sinusitis is one of the most prevalent medical conditions, affecting millions of individuals worldwide, with a significant morbidity and healthcare burden [1]. It manifests as paranasal sinus inflammation and can be acute, subacute, chronic, or recurrent, depending on the persistence and duration of the symptoms [2]. Unilateral sinus disease (USD) is a subset of sinusitis and can occur due to a wide range of conditions, such as tumors, periodontal diseases, fungus balls, and retained foreign bodies [3,4]. However, it is challenging to diagnose due to its non-specific clinical presentations [5]. The USD is characterized by nasal obstruction, postnasal drip or unilateral rhinorrhea, facial pressure or pain, headache, epistaxis on the affected side, or sometimes visual problems [6]. Although many sinonasal diseases begin with symptoms on one side, increasing evidence indicates that the opposite side often becomes affected over time [7]. Therefore, its management is necessary and often involves a combination of medical therapies and surgical interventions, particularly functional endoscopic sinus surgery (FESS), with the aim of restoring aeration by removing the obstructions and drainage in the sinus [8].

The theory behind FESS is the re-establishment of drainage from the ethmoid, maxillary, frontal sinuses, sphenoid, and sinus ventilation and improving the normal functions [9,10]. In addition, FESS is recommended for infectious sinus and inflammatory illness, chronic rhinosinusitis with nasal polyps [11,12]. It is also helpful in the establishment of mucociliary clearance from the dependent sinuses by allowing the ostiomeatal complex to stay patent [9]. Meanwhile, this surgical approach is more appropriate for individuals with acute or chronic sinusitis, and improvement can be observed in over 90% of the procedures [13]. Moreover, compared to bilateral sinus surgery, unilateral sinus surgery showed better improvement [14]. In clinical practice, there has been increasing attention to the potential involvement of the contralateral sinus, even in patients who initially present with unilateral pathology [15,16]. Despite the unilateral presentation of many sinonasal diseases, there is growing evidence to suggest that the contralateral side may not remain unaffected in the long term [17]. Several studies have evaluated unilateral allergic fungal rhinosinusitis for the recurrence rate of uninvolved contralateral sinuses and observed eventual involvement of the non-disease side [18]. Furthermore, mucosal thickening and inflammatory changes in the contralateral sinus are even observed in patients undergoing unilateral maxillary sinus opacification [19]. This has raised important concerns regarding whether prophylactic surgical intervention on the non-diseased side could prevent the progression of the disease or provide additional benefits over time.

The emerging evidence suggests that, despite initial unilateral symptomatology, the contralateral sinonasal cavity may exhibit disease progression over time due to interconnected pathophysiological mechanisms. This raises a clinical dilemma regarding whether prophylactic surgery on the asymptomatic side could pose unnecessary risks. Therefore, there is an urgent need to systematically analyze the evidence and provide clear guidelines for decision-makers. Thus, the present study aimed to systematically evaluate the existing literature on sinus surgery on the non-diseased side in patients with unilateral disease.

This review is also helpful for answering the following questions: (1) What is the impact of sinus surgery on the non-diseased contralateral side in patients with USD?

## Review

Methodology

Study Design

The Preferred Reporting Items for Systematic Reviews and Meta-analysis (PRISMA), a 27-item-based guideline, was used for the transparency and reproducibility of the outcomes [20].

Inclusion and Exclusion Criteria

This study included studies following PIO guidelines, as described in Table 1.

**Table 1:** Inclusion and exclusion criteria for the selection of studies. Abbreviations: P: Population, I: Intervention: O: Outcome: RCT: Randomized Controlled Trial, FESS: Functional Endoscopy Sinus Surgery, USD: Unilateral Sinus Disease

Framework	Inclusion criteria	Exclusion criteria
P	Patients with non-diseased contralateral side with USD	Patients with diseased contralateral side with USD and studies on primary bilateral sinus disease
I	FESS	Non-FESS
O	Indications, clinical outcomes, recurrence rates, and complications	Studies with incomplete outcomes data
Study design	RCTs and non-RCTs (retrospective, cohort, case-control, observational, case study)	Animals, cadaveric studies, reviews, editorials, letters, commentaries, conference papers, abstracts, preprints, case study
Language	English	Non-English

Search Strategy

Different electronic databases, such as PubMed, Scopus, Cochrane Library, ScienceDirect, and Google Scholar, were searched till February 2025. Different keywords, such as (“Sinus surgery” OR “functional endoscopic sinus surgery” OR “endoscopic sinus surgery”) AND (“unilateral sinus disease” OR “unilateral chronic rhinosinusitis” OR “unilateral rhinosinusitis” OR “unilateral sinusitis”) AND (“non-diseased sinus” OR “opposite side” OR “contralateral sinus”), were used (see appendices, Supplementary Table 1).

Studies' Screening and Selection Process

**Figure 1:**
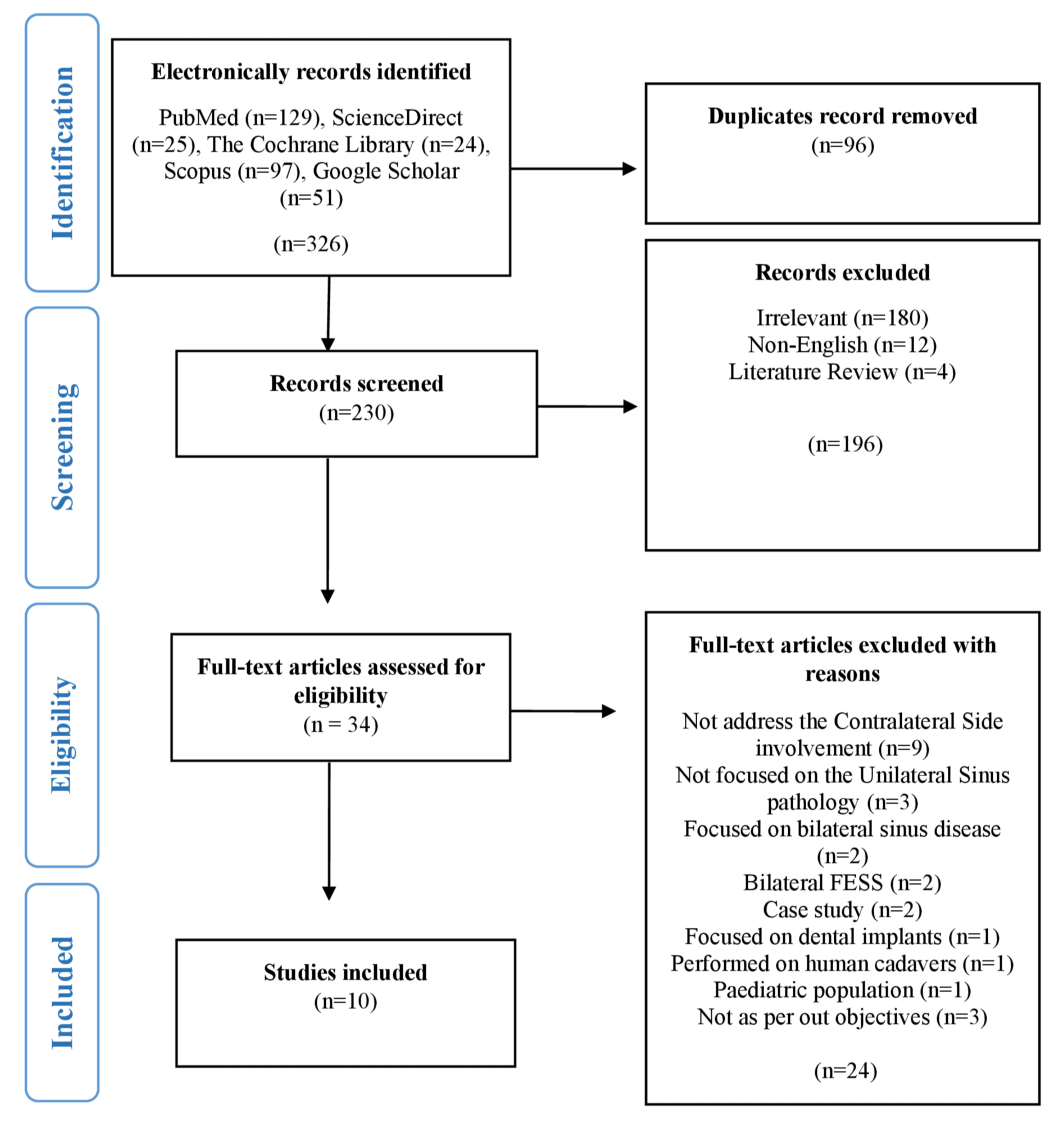
PRISMA flow chart for the selection of the studies. PRISMA: Preferred Reporting Items for Systematic Reviews and Meta-Analysis

Screening of the studies was performed using a four-stage screening process of the PRISMA flow chart. Initially, 326 studies were identified from different bases in the first stage and retrieved using EndNote X9 referencing software. Ninety-six duplicate studies were removed, and the remaining 230 studies were moved to the second stage for the screening process. During this process, each study title and abstract was screened, and 34 studies were found according to our aim, and then they were moved to the third stage of the full-text assessment phase. In this phase, each study was fully assessed according to the inclusion/exclusion criteria set for the selection of studies. After the assessment, 10 studies were moved to the included stage, and 24 studies were excluded, with reasons explained in Figure 1. These 10 selected studies were further analyzed qualitatively. This whole process of screening and study selection was performed by two independent authors, and the discrepancy was resolved by contacting a third author.

Data Extraction

Two independent authors used a predefined data extraction form, and all relevant information was extracted, which included different variables:

Study characteristics: Authors, year, country, study design, and sample size

Participants' characteristics: Gender and age

Disease characteristics: Duration and stage

Intervention characteristics: Indication

Outcomes: Symptom relief, recurrence rate, complications, follow-up, and conclusion

Reporting Assessment of the Observational Studies

Strengthening the Reporting of Observational Studies in Epidemiology (STROBE) was used for the methodological quality assessment. STROBE, a 22-item-based assessment framework, was used for the evaluation of studies in the domain of study design, justification of sample size, bias control, statistical methods, and outcome reporting. Each study was assessed by two independent reviewers and rated as low, moderate, or high quality based on assessed domains [21].

Methodological Quality Assessment

Methodological quality assessment of observational studies (cohort, retrospective, prospective, and case-control studies) was assessed by Risk of Bias in Non-randomized Studies-Interventions (ROBINS-I), and responses were categorized as low RoB, high RoB, and some concerns [22]. Further, for visualization of the outcomes, Robvis, a web-based tool, was used [23]. Two independent reviewers conducted the methodological quality assessment.

GRADE Framework for Certainty of Evidence

The Grading of Recommendations, Assessment, Development and Evaluation (GRADE) was used to ensure the certainty of the evidence. This process explicitly included all important and critical outcomes of the review. The main domains used for certainty were RoB, inconsistency, evidence indirectness, impression, and publication bias [24].

Data Synthesis

A narrative synthesis was performed, summarizing the basic information of the studies and key findings. Findings were organized according to the predefined categories in the Microsoft Excel sheet (Microsoft® Corp., Redmond, WA) for constructing tables and graphs.

Results

Study Distribution

**Figure 2:**
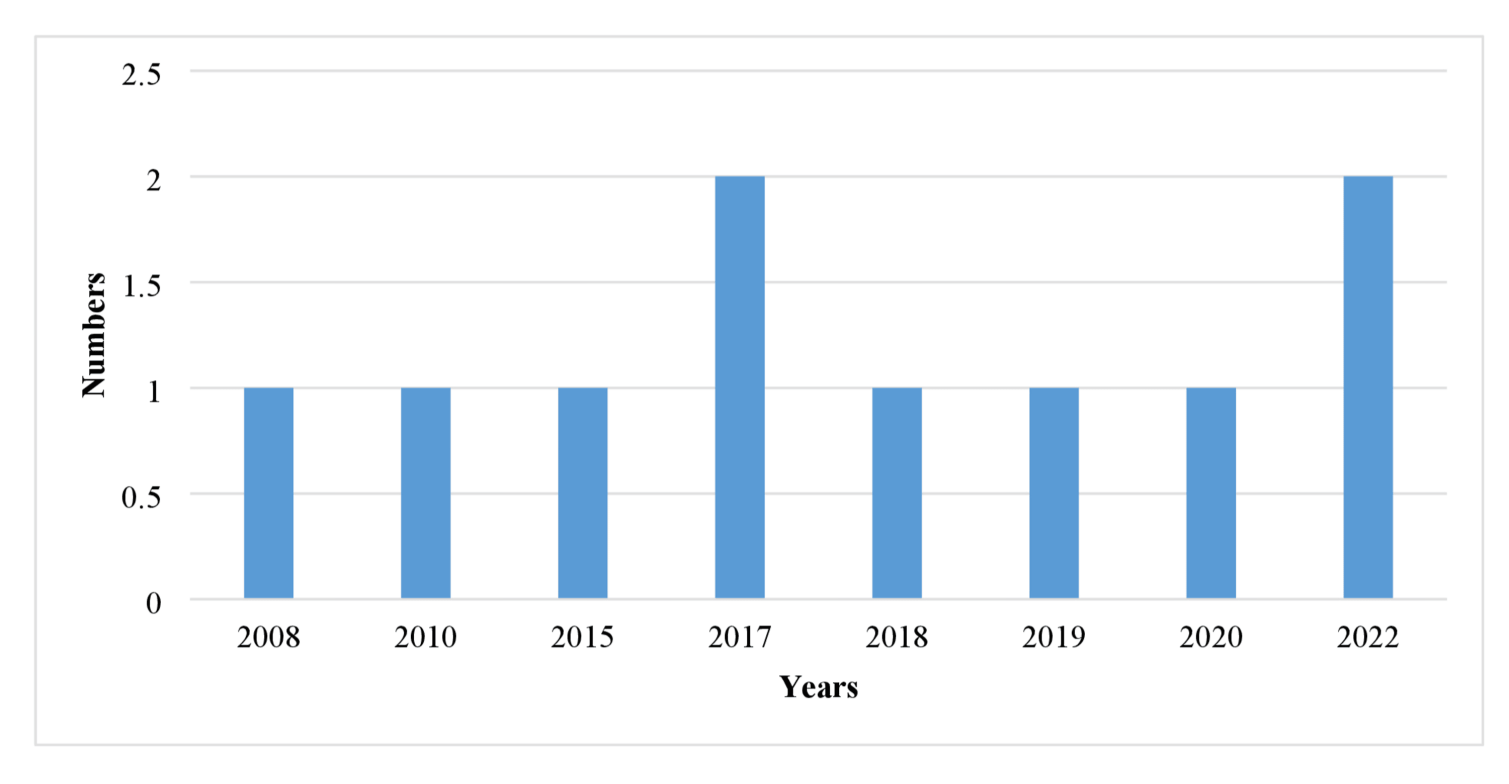
Studies are distributed all over the world with a number of publications.

The majority of the studies were published in 2017 (2/10, 20%) and 2022 (2/10, 20%), while each study (1/10, 10%) was published in 2008, 2010, 2015, and 2018-2020. In addition, major contributions to the publication of these studies were from South Korea (3/10, 30%) and the USA (2/10, 20%). Germany, India, Saudi Arabia, Morocco, Egypt, and Taiwan also contributed to these publications (Figure 2).

General Characteristics

All of the studies evaluated the impact of surgery on the diseases associated with sinus, which are clearly stated in the objectives of each study. All of the studies were non-randomized controlled trials (RCTs) and followed retrospective [16,25-28], prospective [29-32], and retrospective case control [15]. These studies were performed in a range of settings, including academic medical centers, hospitals, and tertiary healthcare centers across different regions (Table 1). Similarly, variation in the sample was observed, with a minimum of 42 and a maximum of 288 sample size [25,32]. Similarly, a wide range of participant ages was observed, ranging from 27.5 to 64.4 years [15,31]. Gender distribution is another important characteristic, with most of the studies showing female participants at a higher rate [15,16,29,31]. The duration of follow-up varies considerably, ranging from three months to 4.1 years [33,34]. Meanwhile, the STROBE analysis indicates the reporting quality of the studies. The reporting percentages vary across the studies, ranging from 27% to 91% [15,27], as described in Table 2.

**Table 2:** Summary of the general characteristics of the studies and participants. Abbreviations: STROBE: Strengthening the Reporting of Observational Studies in Epidemiology, CRS: Chronic Rhinosinusitis, FESS: Functional Endoscopic Sinus Surgery, ESS: Endoscopic Sinus Surgery, MMA: Middle Meatal Antrostomy, AFRS: Allergic Fungal Rhinosinusitis, SNOT-22: Sino-Nasal Outcome Test-22, NA: Not Available, M: Male, F: Female, ORL: Otolaryngology, H&N: Head & Neck, USD: Unilateral Sinus Disease, NP: Nasal Polyp, QOL: Quality of Life

Authors	Objectives	Study Design	Setting/Bed	Sample Size	Age (Year)	Gender (M:F)	Follow-up (Months)	STROBE (%)
Lee, 2008 [26]	To evaluate the clinical features, diagnosis, pathology, and computed tomography findings in patients who had undergone sinus surgery for unilateral sinus diseases	Retrospective	Soonchunhyang University Bucheon Hospital	121	38.9	79:42	NA	64%
Lee et al., 2010 [30]	To evaluate subjective and objective surgical outcomes between patients with unilateral and bilateral CRS accompanying NP	Prospective	NA	Unilateral: 23, Bilateral: 158	Unilateral: 42.8, Bilateral: 45.1	Unilateral: 16:7, Bilateral: 109:49	6	64%
Troeltzsch et al., 2015 [28]	To correlate demographic, anamnesis, clinical and radiological data with histological and microbiological results and intraoperative findings, to determine the underlying reason for sinusitis unambiguously	Retrospective cohort	University hospital	174	52.7	1.4:1 ratio	NA	55%
AlQahtani et al., 2017 [15]	To evaluate the recurrence forms of unilateral AFRS as well as to study the possible predictor factors of developing the disease on the contralateral side	Retrospective case-control study	ORL/H&N departments of nine national tertiary care centers	68	27.5 ± 11.5	25:43	16.9	91%
Mielcarek-Kuchta et al., 2017 [27]	The analysis of USD in the group of patients who underwent FESS at the secondary referral center	Retrospective	Secondary referral center (Provincial Hospital)	83	NA	48:35	2 years	27%
McCoul, 2018 [31]	To identify defining characteristics in patients who manifested an isolated, dysfunctional sinus in the setting of prior sinus surgery	Prospective case series	Tertiary rhinology practice	113	64.4 ± 12.3	35:78	16	41%
Laababsi et al., 2019 [29]	1. Report and compare outcome of patients with unilateral CRSsNP and bilateral CRSsNP undergoing FESS. 2. Evaluate the impact of SNOT-22 domains and especially the hypothesis of the unilateral nature of CRSsNP to predict the QOL outcome after FESS.	Prospective cohort	Hospital based	66	Unilateral: 38.76, Bilateral: 39.57	Unilateral: 19:26, Bilateral: 12:9	12	77%
Kim et al., 2020 [25]	To assess facets of MMA in patients undergoing ESS with diseased maxillary sinuses.	Retrospective	Soonchunhyang University medical records	288	31-60	192:96	6	77%
Gulati et al., 2022 [16]	To explore the degree to which patients undergoing unilateral ESS experience post-operative contralateral sinonasal symptoms and determine risk factors for contralateral symptomatology following unilateral ESS	Retrospective	Northwestern Memorial Hospital	97	55	43:54	NA	68%
Yang et al., 2022 [32]	To investigate the prevalence of abnormal nasality in patients with unilateral rhinosinusitis and their nasality outcomes following FESS	Prospective	Kaohsiung Chang Gung Memorial Hospital	42	49.9	21:21	12	68%

Outcomes

Table 3 summarizes the pathological and surgical outcomes of the selected studies associated with FESS for mostly CRS and other associated conditions. Different pathologies were identified, including CRS alone, CRS with NP, ACP, fungus ball, benign tumor (including mucoceles), malignant neoplasia [26], NP alone, acute or chronic unilateral sinusitis [28,30], unilateral allergic fungal rhinosinusitis (AFRS) [15], and chronic, persistent postnasal drainage with a unilateral mucopurulent exudate [31]. They assessed the effectiveness of FESS (unilateral or bilateral), as described in Table 2. Most of the studies reported significant improvement in patients, with a high success rate in improving symptoms such as nasal obstruction and postnasal drainage, with few studies reporting a recurrence of the disease, for instance, 55.8% of patients’ overall recurrence with 36.8% exclusive contralateral involvement [15]. Similarly, 10 patients were reported with recurrent maxillary sinusitis due to stenosis at the middle meatal antrostomy (MMA) site, occurring within six months after surgery [25]. Similarly, two patients showed recurrent unilateral chronic rhinosinusitis (CRS) following surgery [32]. In addition, medical management typically involved β-lactam antibiotics, decongestants, mucolytics, and oral or topical nasal steroids [15,26,27,30,32]. Moreover, unilateral sinus pathologies often had better surgical outcomes compared to the bilateral cases. Meanwhile, contralateral symptoms developed after FESS, as indicated in Table 3.

**Table 3:** Summary of pathological and surgical outcomes. Abbreviations: CRS: Chronic rhinosinusitis, NP: Nasal Polyp, AFRS: Allergic Fungal Rhinosinusitis, SNOT: Sino-Nasal-Outcome-Test, CRS, FESS: Functional Endoscopic Sinus Surgery, ESS: Endoscopic Sinus Surgery, USD: Unilateral Sinus Disease, MMA: Middle Meatal Antrostomy, QOL: Quality of Life, SD: Standard Deviation, NA: Not available

Author	Pathology	Surgery (Unilateral/Bilateral)	Recurrence	Timeframe (Months)	Medical Management	Key Findings	Conclusion
Lee, 2008 [26]	CRS, CRS with NP, antrochoanal polyp, fungus ball, benign tumor (including mucoceles), and malignant neoplasia	FESS with or without polypectomy (unilateral)	NA	NA	b-lactam antibiotics, decongestants, mucolytics, and oral or topical nasal steroids	A much higher percentage of unilateral pathology than previously reported, and the majority was benign (92.6%)	This study observed a much higher percentage of unilateral sinus pathology
Lee et al., 2010 [30]	CRS and NP	FESS with nasal polypectomy (unilateral and bilateral)	NA	NA	Antibiotics, mucolytics, topical nasal steroid spray, and a 10-day course of systemic steroids (a single daily dose of prednisolone, 20 mg)	In the unilateral group, 20 patients (87.0%) had good outcomes, compared with 102 patients (64.6%) in the bilateral group	Unilateral CRS with NP showed more favorable objective surgical outcomes than bilateral diseases
Troeltzsch et al., 2015 [28]	Acute or chronic unilateral sinusitis	FESS (unilateral)	NA	NA	NA	No differences in the clinical appearance of the disease with respect to its etiology	Odontogenic causes for maxillary sinusitis must be considered, especially in unilateral cases
AlQahtani et al., 2017 [15]	Unilateral AFRS	FESS (unilateral)	Recurrence rate: 55.8% (14 patients with exclusive contralateral involvement (36.8%)	16.9	Post-operative use of systemic steroids	Contralateral side involvement was occurred	The non-diseased sinuses should be involved in the routine endoscopic examination and post-operative treatment.
Mielcarek-Kuchta et al., 2017 [27]	Chronic sinusitis, CRS, mycosis, non-malignant tumors, choanal polyp	FESS (unilateral)	No recurrence	NA	Aggressive steroid therapy	USD must be always suspected of malignant degeneration until proven otherwise	Endoscopic sinus surgery with the use of angled scopes allows for the removal of even very extensive lesions
McCoul, 2018 [31]	Chronic, persistent postnasal drainage with a unilateral mucopurulent exudate	Prior sinus surgery	NA	NA	NA	The mean (SD) osteitis score for the condemned sinus was 2.7 (1.1) compared to 1.2 (0.4) on the contralateral side (P<0.01)	The condemned sinus is a distinct entity that may represent a sequela of previous non–mucosal-sparing surgery. An association with hyperostosis is observed
Laababsi et al., 2019 [29]	CRS	FESS (unilateral and bilateral)	NA	NA	NA	A higher significant improvement was observed between preoperative and post-operative SNOT-22 scores in unilateral CRSsNP group [37.13 ± 9.307 versus 14.11 ± 8.531] and in bilateral CRSsNP group [41.76 ± 6.949 versus 18.57 ± 8.495]	FESS improves all domains of QOL
Kim et al., 2020 [25]	Polyp, fungal ball, allergic rhinitis	FESS	Recurrent maxillary sinusitis due to MMA site stenosis occurred in 10 patients	6	NA	Most had unilateral sinusitis, and stenosis was observed within 6 months post-operatively	Conservative trimming, meticulous dressing, and removal of sinus crust and granulation tissue near the MMA site should be performed in patients with MMA site stenosis
Yang et al., 2022 [32]	Unilateral CRS (CRS with NP, CRS without polyp, fungal sinusitis, odontogenic sinusitis,	FESS (unilateral)	2 patients had recurrence	NA	Intranasal corticosteroids for at least 2 months and oral steroids or antibiotics (depending on their condition) for 2 weeks	One-third of unilateral CRS patients had abnormal nasality preoperatively and significant improvement following FESS	Although only one side of the nasal airway was involved, one-third of the patients reported abnormal nasality
Gulati et al., 2022 [16]	CRS	FESS (unilateral)	NA	NA	NA	24% of patients reported contralateral congestion, a median of 24 months post-ESS, and more than 10% of patients reported other contralateral symptoms including swelling, rhinorrhea, difficulty breathing, and hyposmia post-ESS	Patients who have unilateral ESS for CRS may experience long-term contralateral symptoms. Having a septoplasty did not affect contralateral symptoms

Methodological Quality Assessment

**Figure 3:**
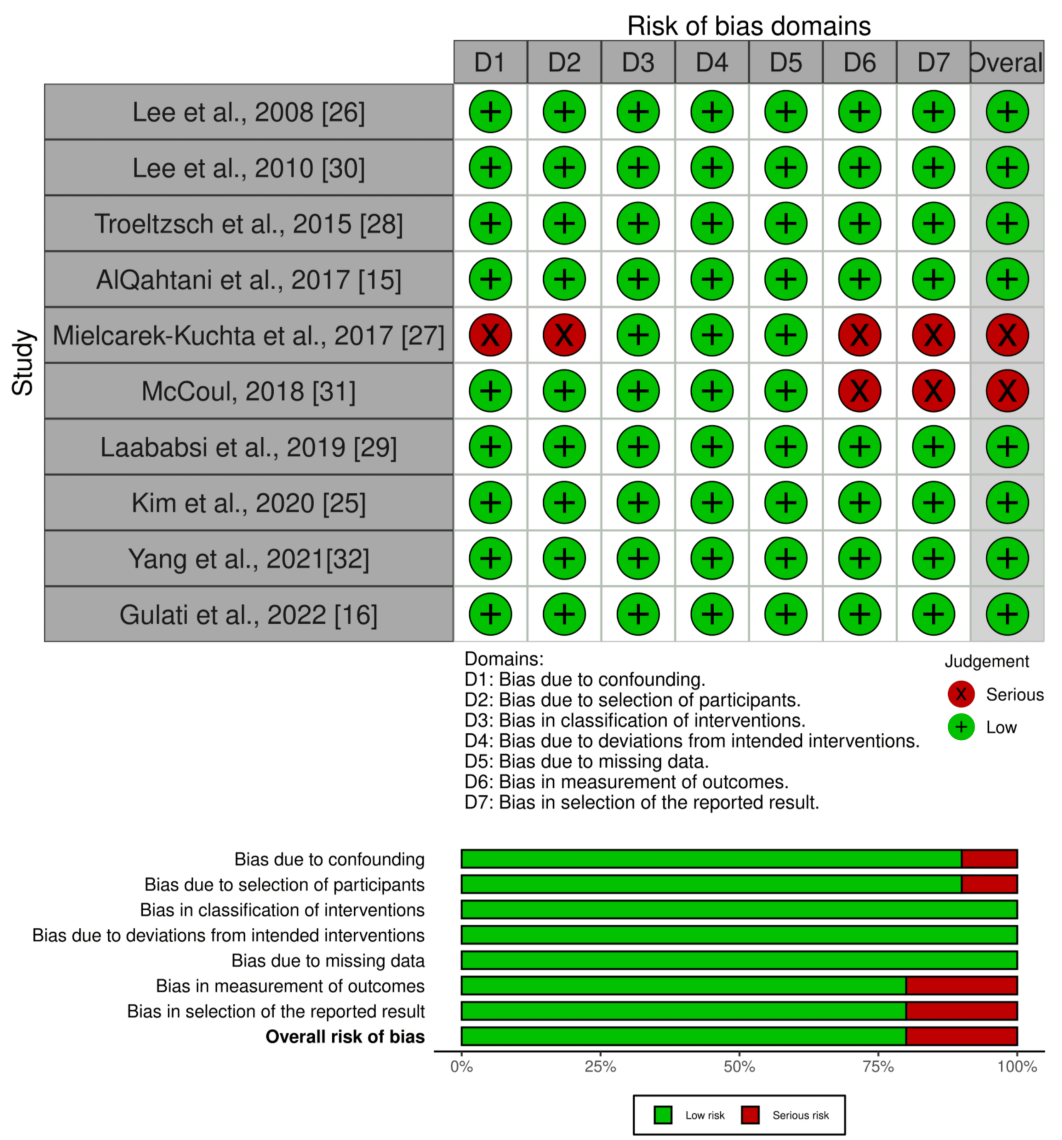
Methodological quality assessment of non-RCTs. Abbreviations: RCTs: Randomized Controlled Studies

Overall, eight studies (80%) had low RoB, while two studies (20%) had serious RoB, particularly in the domain of bias due to confounding variables, selection of participants, measurement of outcomes, and selection of the reported results [27,31], as described in Figure 3.

Certainty of Evidence

In the domain of methodological limitations, no serious concern was observed, as most of the studies (80%) had low RoB. Similarly, STROBE was performed for the reporting analysis, and most of the studies had a score of >50% and showed no serious concern. Furthermore, the majority of the studies explained the selection process for the patients; therefore, imprecision was also of no serious concern. Moreover, both negative and positive outcomes were reported in the studies; thus, there was no publication bias (Table 4).

**Table 4:** Summary of the certainty of evidence using the GRADE framework. Abbreviations: RoB: Risk of Bias, STROBE: Strengthening the Reporting of Observational Studies in Epidemiology, GRADE: Grading of Recommendations, Assessment, Development and Evaluation

GRADE Domain	Judgement Description	Concerns	Certainty of Evidence
Methodological limitations	Overall, two studies had serious risk of bias, one study showed serious RoB in four domains (bias due to confounding variables, selection of participants, measurement of outcomes, and selection of reporting results) [27], and one study demonstrated serious RoB in 2 domains (measurement of outcomes, and selection of reporting results) [31]. Even two studies had methodological limitations, however, 8 studies (80%) had low RoB. Meanwhile, only retrospective studies were included.	Some concerns	Low
Indirectness	Most of the studies reported the characteristics of patients, intervention, and clinical variables. For the reporting analysis, STROBE was performed which indicated that most of the studies reported >50% of the items.	Not serious	
Imprecision	Overall, acceptable number of patients were included in the selected studies. Most of the studies clearly define the selection criteria for the patients.	Not serious	
Inconsistency	Both significant and non-significant outcomes were reported.	Not serious	
Publication bias	Even the funnel plot was not constructed for publication bias, however, we did not find any publication bias observed as both improvement and no change outcomes were reported.	Not suspected	

Discussion

Current clinical practice advocates that the extent of FESS corresponds closely to the anatomical and pathological extent of the disease. Performing bilateral surgery in case of isolated unilateral involvement may result in unnecessary morbidity without clear clinical benefit. However, unilateral sinus surgery increases the risk of recurrence or persistent symptoms due to incomplete treatment. The main focus of this review was to evaluate the role of sinus surgery on the non-diseased contralateral side in patients with USD. All 10 included studies were non-RCT with the following retrospective, prospective, and retrospective, case-control study designs. These studies were conducted in different settings, including academic medical centers, hospitals, and tertiary care centers. The participants’ ages ranged from 27.5 to 64.4 years, with a higher ratio of female participants. There is a higher variation in the patient’s follow-up from three months to 4.1 years.

During the FESS treatment option, different pathological conditions were identified, including CRS alone, CRS with NP, ACP, fungus ball, benign tumor, malignant neoplasia, NP alone, acute or chronic unilateral sinusitis, AFRS, and chronic, persistent postnasal drainage with a unilateral mucopurulent exudate. The reason for these pathologies is not apparent; however, they may be a part of natural disease progression. These pathologies have a detrimental impact on the quality of life. Similarly, an animal model study reported that unilateral sinus surgery may cause inflammation and be responsible for contralateral sinus involvement [35]. Another study reported the frequent postoperative development of contralateral disease in unilateral AFRS without the involvement of the original operated side. These findings need special attention to patient management, follow-up, and counseling [36]. In addition, these findings highlight that unilateral FESS may lead to long-term symptoms on the non-diseased contralateral side, which underscores the significance of patient counseling related to adverse contralateral effects.

Most of the studies reported significant improvement in patients, with a high success rate in improving symptoms such as nasal obstruction and postnasal drainage. The success of the FESS surgical procedure depends upon various parameters, including disease severity, extent of surgery, patient’s general characteristics (smoking), and adherence to postoperative care [37]. However, few studies have reported a recurrence of the disease, and it remains a clinical consideration in the post-operative management of USD. A recent review investigated the recurrent inflammation of contralateral sinuses in unilateral AFRS, suggesting that the non-diseased contralateral side should be involved in routine endoscopic examination, and postoperative medical or surgical therapy may improve the recurrence rate and improve inflammatory control [18]. Furthermore, in another retrospective study, 136 cases of CRS with or without nasal polyps underwent ESS, and during the 24.8 months of follow-up, four (25%) patients developed AFRS on the contralateral side [36]. Similarly, another study observed 25% (8 patients) post-operative recurrence rate; among these eight patients, three had unilateral, and three had bilateral disease [38]. Similarly, a retrospective analysis of 118 patient records demonstrated that a significant number of patients (71, 60%; p<0.005) had severe polyposis after ESS [39]. Meanwhile, in the present study, the mean follow-up time was low, or it did not mention that future studies with longer follow-up might have a higher rate of contralateral involvement in patients. Moreover, for recurrence, another assumption may be the transfer of pathogens, such as fungal antigens, to the contralateral side from the infected side due to the intraoperative or postoperative irrigations. Elevated levels of interleukin (IL)-5 were also associated with increased risk of recurrence and revision surgery [40]. Several risk factors, such as smoking, asthma, presence of allergy, septal deviation, aspirin intolerance, peripheral eosinophilia, T2 profile, and intense opacification, also play a contributing role in the recurrence [41].

In the present review, medical interventions typically involved β-lactam antibiotics, decongestants, mucolytics, and oral or topical nasal steroids. These medical treatments reduce inflammation, prevent recurrence, and inhibit the progression of disease in contralateral sinuses. Moreover, unilateral sinus pathologies often had better surgical outcomes than bilateral ones. Endoscopic examination of the non-diseased contralateral side is critical before and after FESS in patients with USD to observe the development of initial signs and symptoms of disease. In addition, regular monitoring and treatment plans for the non-diseased contralateral side are crucial to inhibit disease progression and achieve optimal patient outcomes.

Strengths and Limitations

The strengths of the present review include a comprehensive literature search from different electronic databases, the inclusion of articles that explore sinus surgery on the non-diseased contralateral side, independent methodological quality, level of evidence, risk of bias assessment, and rigorous data synthesis analysis. Additionally, this review has a few limitations. During the literature search from different databases, we have restricted to English-language studies, which may cause bias and affect the generalizability of outcomes. Another important limitation of the present review is the inclusion of retrospective and case reports, which generate a low level of evidence. Meta-analysis was not performed due to the unavailability of uniform data. In the future, longitudinal and multicentre studies with large sample sizes would be helpful in examining the role of sinus surgery on the non-diseased contralateral side in patients with USD. In addition, it would markedly strengthen the study by incorporating prospective, endotype-guided, multicentric RCTs with rigorous contralateral surveillance metrics, allowing a more definitive interrogation of prophylactic or pre-emptive bilateral surgical strategies.

## Conclusions

This review examined the effect of sinus surgery on the non-diseased contralateral side in patients with USD. Current findings demonstrated that unilateral FESS might lead to long-term symptoms on the non-diseased contralateral side, suggesting the importance of patient counseling related to adverse contralateral effects. Additionally, the recurrent inflammation of the contralateral sinuses was reported, suggesting that the contralateral side should be involved in routine endoscopic examination and medical interventions may improve the recurrence rate and improve inflammatory control on the contralateral side. Furthermore, longitudinal and multicenter studies with large sample sizes should be conducted to validate the current findings.

## Appendices

**Table 5:** A literature search from different databases (Supplementary Table 1).

Databases	Key words
PubMed	((("paranasal sinuses"[MeSH Terms] OR ("paranasal"[All Fields] AND "sinuses"[All Fields]) OR "paranasal sinuses"[All Fields] OR "sinus"[All Fields] OR "sinus s"[All Fields]) AND ("surgical procedures, operative"[MeSH Terms] OR "general surgery"[MeSH Terms])) OR "functional endoscopic sinus surgery"[All Fields] OR "FESS"[All Fields] OR "endoscopic sinus surgery"[All Fields]) AND ("unilateral sinus disease"[All Fields] OR "USD"[All Fields] OR "unilateral chronic rhinosinusitis"[All Fields] OR "unilateral rhinosinusitis"[All Fields] OR "unilateral sinusitis"[All Fields]) ((("paranasal sinuses"[MeSH Terms] OR ("paranasal"[All Fields] AND "sinuses"[All Fields]) OR "paranasal sinuses"[All Fields] OR "sinus"[All Fields] OR "sinus s"[All Fields]) AND ("surgical procedures, operative"[MeSH Terms] OR "general surgery"[MeSH Terms])) OR "functional endoscopic sinus surgery"[All Fields] OR "FESS"[All Fields] OR "endoscopic sinus surgery"[All Fields]) AND (("non-diseased"[All Fields] AND ("paranasal sinuses"[MeSH Terms] OR ("paranasal"[All Fields] AND "sinuses"[All Fields]) OR "paranasal sinuses"[All Fields] OR "sinus"[All Fields] OR "sinus s"[All Fields])) OR "opposite side"[All Fields] OR "contralateral sinus"[All Fields])
ScienceDirect	(“Sinus surgery” OR “functional endoscopic sinus surgery” OR “endoscopic sinus surgery”) AND (“unilateral sinus disease” OR “unilateral rhinosinusitis” OR “unilateral sinusitis”) AND (non-diseased sinus OR “opposite side” OR “contralateral sinus”)
Cochrane Library	((“Sinus surgery” OR “functional endoscopic sinus surgery” OR “FESS” OR “endoscopic sinus surgery”)):ti,ab,kw AND ((“unilateral sinus disease” OR “USD” OR “unilateral chronic rhinosinusitis” OR “unilateral rhinosinusitis” OR “unilateral sinusitis”)):ti,ab,kw ((“Sinus surgery” OR “functional endoscopic sinus surgery” OR “FESS” OR “endoscopic sinus surgery”)):ti,ab,kw AND ((non-diseased sinus OR “opposite side” OR “contralateral sinus”)):ti,ab,kw
Scopus	(“Sinus surgery” OR “functional endoscopic sinus surgery” OR “FESS” OR “endoscopic sinus surgery”) AND (“unilateral sinus disease” OR “USD” OR “unilateral chronic rhinosinusitis” OR “unilateral rhinosinusitis” OR “unilateral sinusitis”) (“Sinus surgery” OR “functional endoscopic sinus surgery” OR “FESS” OR “endoscopic sinus surgery”) AND (“non-diseased sinus” OR “opposite side” OR “contralateral sinus”)
Google Scholar	(“Sinus surgery” OR “functional endoscopic sinus surgery” OR “endoscopic sinus surgery”) AND (“unilateral sinus disease” OR “unilateral chronic rhinosinusitis” OR “unilateral rhinosinusitis” OR “unilateral sinusitis”) AND (“non-diseased sinus” OR “opposite side” OR “contralateral sinus”)
